# Hypoplastic Left Heart Syndrome: About a Postnatal Death

**DOI:** 10.3390/diagnostics13050821

**Published:** 2023-02-21

**Authors:** Pasquale Giugliano, Paola Ciamarra, Mariavictoria De Simone, Alessandro Feola, Pierluca Zangani, Carlo Pietro Campobasso, Gelsomina Mansueto

**Affiliations:** 1 A.O. Sant’Anna e San Sebastiano, 81100 Caserta, Italy; 2 Department of Experimental Medicine, University of Campania “Luigi Vanvitelli”, 80138 Naples, Italy; 3 Department of Advanced Medical and Surgical Sciences (DAMSS), University of Campania “Luigi Vanvitelli”, 80138 Naples, Italy

**Keywords:** hypoplastic left heart syndrome, respiratory insufficiency, shock, cardiogenic, autopsy

## Abstract

Background: Hypoplastic left heart syndrome (HLHS) is a congenital heart disease that is associated with high mortality rates in the early neonatal period and during surgical treatments. This is mainly due to missed prenatal diagnosis, delayed diagnostic suspicion, and consequent unsuccessful therapeutic intervention. Case report: twenty-six hours after birth, a female newborn died of severe respiratory failure. No cardiac abnormalities and no genetic diseases had been evidenced or documented during intrauterine life. The case became of medico-legal concern for the assessment of alleged medical malpractice. Therefore, a forensic autopsy was performed. Results: the macroscopic study of the heart revealed the hypoplasia of the left cardiac cavities with the left ventricle (LV) reduced to a slot and a right ventricular cavity that simulated the presence of a single and unique ventricular chamber. The predominance of the left heart was evident. Conclusions: HLHS is a rare condition that is incompatible with life, with very high mortality from cardiorespiratory insufficiency that occurs soon after birth. The prompt diagnosis of HLHS during pregnancy is crucial in managing the disease with surgery.

## Figures

**Figure 1:**
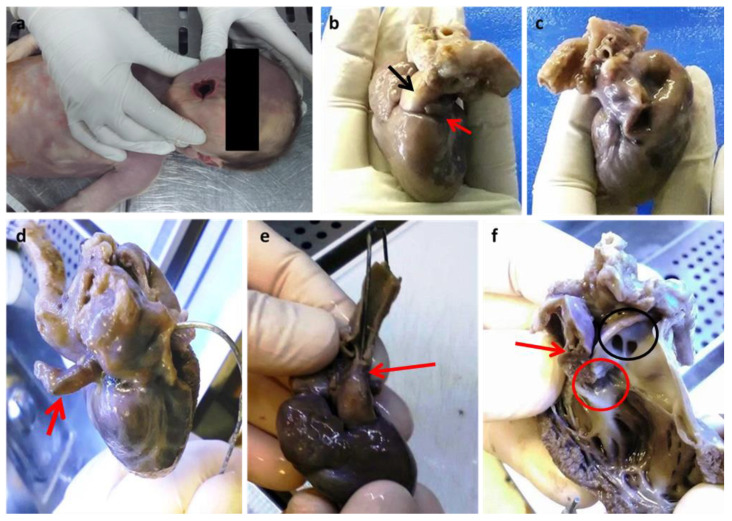
(**a**) Cyanosis of the buccal rim. Anterior face (**b**) and posterior face (**c**) of the heart. The red arrow points to a small and virtual auricle-like left atrial cavity (**b**) which is better highlighted by the introduction of a probe (**d**); the black arrow indicates the emergence cone of the predominant pulmonary trunk which is predominant (**b**). Evidence of hypoplastic aorta after rotation of the heart towards the posterior face (**e**). Prevalence of the right ventricle (**f**): the red arrow indicates a small residual left ventricular cavity with a small residual sept; the red circle is the only identified right valve; the black circle indicates the emergence of the right and left pulmonary vessels above the emergence of the common trunk. Dissection of the right anterior ventricular wall and pulmonary trunk following the outflow route. (Images from Mansueto’s forensic archive). The missing intrauterin diagnosis of fetal defects could be the cause of unexpected newborn deaths. HLHS is a syndrome characterized by multiple congenital cardiac structural abnormalities, which was first described by Lev in 1952 [1]. Currently, the mortality rate from HLHS is approximately 2–3% of all congenital heart diseases, with 23% of deaths within the first week and 15% within the first month [1,2]. Treatment has enabled infants with HLHS to survive beyond the first decade of life, with a reported 15-year survival of 48% and significant mortality during the first year of life [2,3,4]. An alteration of cardiogenesis is now clearly identified as the cause of the underdevelopment of the left heart, which is associated in most cases with mitral and ascending aortic anomalies in HLHS. Consistent with multifactorial aetiology and impaired cardiogenesis, HLHS, therefore, represents a syndrome in which the phenotypic spectrum can vary and be more or less faithful to the identified entities. In addition to defective cardiogenesis, left ventricular hypoplasia can also be explained by decreased blood flow during development. Both of these factors can coexist [5,6]. During intrauterine life, the vascular system of the fetus reaches a functional balance supported by maternal-fetal circulation. The maternal-fetal balance is lost after birth, after the ductus arteriosus and foramen ovale close, resulting in rapid cardiorespiratory failure. Therefore, the early diagnosis of HLHS is essential for timely medical therapy and for the choice of surgical therapy consisting of heart transplantation and/or palliative procedures. We describe a case of HLHS-diagnosed post-mortem. A female newborn at 38 weeks of gestational age (birth weight of 2680 g; an Apgar score of 8/10/10) from a 37-year-old mother (multipara without risk factors or pathologies in the anamnesis) showed respiratory failure twenty-six hours after birth. Due to poor clinical condition (oxygen saturation 44%; metabolic acidosis), she underwent mechanical ventilation, intravenous administration of bicarbonate solution, and the administration of prostaglandins for suspected congenital heart disease. Unfortunately, despite cardiopulmonary resuscitation, cardiogenic shock occurred with death. Since no maternal disease or intrauterine heart defect was diagnosed during pregnancy, the question of a diagnosis with prompt treatment to prevent the fatal outcome was asked. Therefore, an autopsy was performed 5 days after death in accordance with the recommendations on the harmonization of forensic autopsy rules of the Committee of Ministers of the Council of Europe (1999). External examination revealed a female newborn with facies composita without phenotypic traits of genetic syndromes, with cyanotic buccal rim, and with the following growth parameters compatible with 38 weeks of gestational age: (crown-heel length 47 cm (39 +/− 2 w), partial length (head-coccyx) 33.5 cm (37 +/− 3 w), skull circumference 33 cm, chest circumference 31 cm, abdominal circumference 28 cm, foot length 6.5 cm (36 +/− 3), femur length 9 cm (>40 w)). Fetal developmental parameters were oriented for 38 weeks of gestation (brain 320.2 g, thymus 7.8 g, heart 16 g, lungs 39.3 g, spleen 7 g, liver 115 g, kidneys 20 g, adrenal glands 3.4 g, pancreas 2.9 g). Macroscopic observation of the heart showed a prevalence of the right heart with a hypoplastic left heart, which was characterized by left atrium reduction to an auricle-like cavity, LV reduced to a slit, and a right ventricle of increased volume to configure almost a single common ventricular chamber. The pulmonary trunk was clearly evident with the valves, as well as the emergence of the left and right pulmonary arteries. The patency of the foramen ovale, a clearly hypoplastic aorta with coarctation aspects, and a patent ductus arteriosus were also observed.

**Figure 2:**
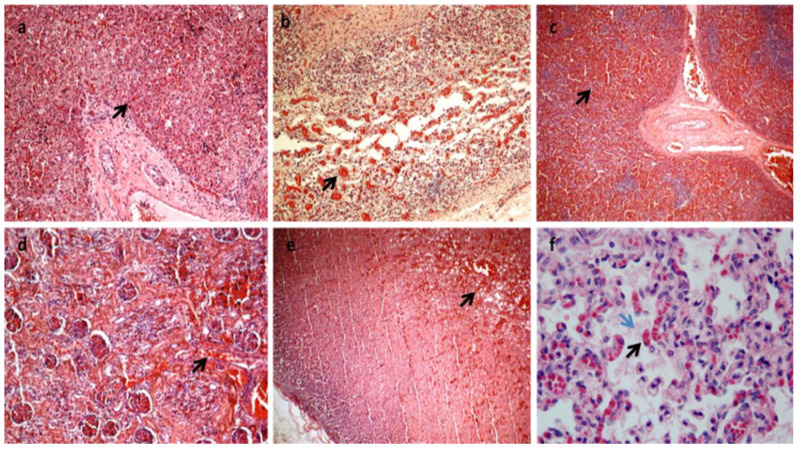
All the organs were macroscopically observed, and samples were taken for each. After formalin fixation and paraffin embedding, sections stained with hematoxylin and eosin were prepared for histological diagnosis [7]. Histology showed multiorgan congestion and lungs in the alveolar-saccular development phase with diffuse congestion with focal aspiration signs (**a**–**f**). In (**a**): congestion and erythrocyte extravasation in hepatic sinusoids (black arrow) (H&E×10). In (**b**): congestion and erythrocyte extravasation in the bowel wall (black arrow) H&E×20). In (**c**): spleen congestion (black arrow) (H&E×4). In (**d**): kidney congestion (black arrow) (H&E×20). In (**e**): adrenal gland congestion (black arrow) (H&E×10). In (**f**): Lungs diffuse congestion (black arrow) with focal intra-alveolar eosinophilic material due to aspiration (blue arrow) (H&E×63 magnification). (Images from Mansueto’s forensic archive). HLHS is a rare type of congenital heart syndrome. Due to the great number of elective terminations of pregnancy and the spontaneous abortion of affected fetuses, the reported overall incidence is likely underestimated. HLHS is not related to maternal age, ethnicity, or geographical factors. Seven in ten patients are male, and 13.5% of siblings of HLHS patients have some form of congenital heart disease, suggesting a complex relationship with multiple genetic factors [2,6,8]. HLHS is a spectrum of cardiac malformations characterized by a hypoplastic left heart system with atresia, stenosis, or hypoplasia of the mitral and/or aortic valves and hypoplasia of the ascending aorta and arch. In addition to the anomalies of the left heart in HLHS, the cardiac atria and extrapericardial aorta are frequently abnormal, thus constituting a very multifaceted problem that is difficult to classify in a single and only phenotype [6,9,10]. This problem becomes even greater if we consider the existence of single ventricular cardiac anomalies that are associated with other syndromes and if we consider that, in practice, a reduction in the LV to "slit" is quite rare and can be confusing. In addition, the phenotypic variability becomes more marked if we consider the different degrees to which anomalies can occur. In any case, the left heart is insufficient and fails to sustain systemic cardiac output. Paradoxically, a functional intrauterine diagnosis is easier than an anatomopathological diagnosis if the heart is in the hands of a non-expert. As regards the physiology of the well-being of the newborn with HLHS, in order to maintain adequate systemic and pulmonary circulation, it is necessary that the exchanges between the left and right cardiac systems are open at birth: (1) the patency of the Botallo duct can ensure systemic perfusion from the right ventricle to the aorta; (2) the foramen ovale or an atrial defect can provide adequate mixing of oxygenated and deoxygenated blood. Flow from the right ventricle depends on the relative resistances of the pulmonary and systemic circuits. The newborn with HLHS may have a short asymptomatic period because the arterial duct is non-restrictive and pulmonary arteriolar resistance is relatively high. The clinical conditions can worsen quickly when the ductus arteriosus closes for the physio-logic post-delivery events, and consequentially systemic perfusion decreases while pulmonary blood flow increases. In fact, most deaths from HLHS occur in the first week of life, with the greatest risk on day 3. This short life can be explained by the closure of the arterial duct within the first 72 h of birth in healthy infants. The rapid diagnosis of HLHS at birth with subsequent prostaglandin E1 treatment can avoid cardiogenic shock and respiratory failure. Clinical classification is very important for the correct management of the newborn. The signs commonly seen in the short history of children with HLHS include cyanosis, respiratory distress, cold extremities, and reduced peripheral pulse. Peripheral cyanosis is the most obvious sign of poor blood oxygenation that cannot be resolved simply by administering oxygen. Tachypnea and respiratory distress may also be associated, and heart sounds with a single loud second heart sound reflect the absence of the aortic valve and the presence of pulmonary hypertension [11,12,13]. In our case, the newborn showed an apparent initial adaptation to extrauterine life, as evidenced by the normality of the Apgar score. Evidently, the ductus arteriosus was still patent, which ensured both pulmonary and systemic perfusion. After 26 h of birth, heart failure occurred, and the newborn was placed in an incubator with 50% O_2_. The main effects of this therapeutic treatment were represented by the increase in oxygen saturation by up to 96% but also by the closure of the arterial duct, which worsened the clinical conditions until death. In fact, no patent ductus arteriosus was found during the macroscopic examination of the heart. Currently, prenatal diagnosis of HLHS can be made in approximately 50 to 75% of cases with fetal echocardiography [2,14,15]. Classical forms with a severely hypoplastic LV can be detected at 11–14 weeks but more commonly in mid-gestation at 18–22 weeks during the standard fetal anatomy screening ultrasound [16,17,18,19,20,21,22,23]. A good prenatal diagnosis allows the pregnant woman to be managed optimally in specialized centers and also to evaluate the possibility of the termination of pregnancy and treatment options that include cardiac transplantation, palliative heart surgery, and exclusive palliative care. Therefore, the screening of the first and second trimesters of gestation is very important [4,15,24,25,26,27,28,29,30]. A postnatal diagnosis following the closure of ductus arteriosus frequently leads to cardiovascular collapse and poor systemic perfusion, requiring cardiopulmonary resuscitation [31,32]. Surgical palliative treatments consist of multiple surgical interventions performed in the first few years of life. However, the possibility of surgical treatment raises an ethical dilemma. Palliative care or abortion is reserved for cases associated with other genetic syndromes which do not have a long expectancy of life. Heart transplantation is the least frequent option because of the scarcity of donors in the neonatal period, the long-term immunosuppression of side effects, and the high mortality rate [33]. Primary heart transplantation is usually reserved for HLHS newborns who show a very high risk of undergoing a staged repair [34,35,36]. It is evident that the management of the newborn and the mother is very complex from both a medical and ethical point of view, but it is also very important in the context of professional misconduct, and a good autopsy is essential.

## Data Availability

The data presented in this study are available on request from the corresponding author. The data are not publicly available due to privacy restrictions.
